# Dental Policy Lab 3: towards oral and dental health through partnership

**DOI:** 10.1038/s41415-021-3733-1

**Published:** 2021-12-17

**Authors:** Nigel B. Pitts, J. Tim Newton, Ross Pow, Nicholas Miller, Catherine Mayne

**Affiliations:** 41415112876001 Faculty of Dentistry, Oral & Craniofacial Sciences, King´s College London, Tower Wing, Guy´s Hospital, London, SE1 9RT, UK; 41415112876002 Power of Numbers, Cambridgeshire, UK

## Abstract

The third and last of the successful Alliance for a Cavity-Free Future (ACFF)/King's College London Dental Policy Lab series, held in 2019, focused on outlining how dental and oral health industries could benefit from enabling positive behaviour change in patients and the public, allowing progress towards caries reduction. During a two-day event, experts from across public health, dentists, global multi-national corporations and dental industry start-ups discussed the issue, collaboratively developing ideas around policy, technology, messaging and engagement for change. An analysis of the current trends in oral health laid out how the implications for industry and corporate social responsibility were identified as crucial. The report and accompanying infographic explored in this paper have been well received and acted as a catalyst for future developments in the area.

## Key points

- Many elements must be focused on to successfully influence public and patient behaviour change.
- Prevention-focused practice models must be implemented to ensure patients are provided with optimal preventive interventions, minimising caries risk and the need for future treatment.
- Changes in regulation and public policy must be seen to free up the dental and oral health industries to innovate and push for developments in caries-reducing technology.
- Oral health must be placed on corporate social responsibility agendas to ensure that industry is focused on seeing health improvements.

## Introduction

### Overview

Over 2.8 billion people globally suffer from untreated dental caries, making it the most prevalent chronic disease affecting humans.^1^ It affects over 530 million children worldwide, with prevalence rates in some children as high as 60-90%. Dental caries shares risk factors with obesity and associated non-communicable diseases (NCDs), and is associated with not only poor oral health, but poor general health. The problems caused by caries are largely preventable, yet despite the increase in acceptance and understanding of the caries continuum, its potential reversibility^2^ and the need for an increase in preventive action, the global caries situation has barely improved over the last 30 years.^3^ In order to see a marked improvement, change must be addressed not only by patients and caregivers, but also by the oral health and dental industries, to encourage improved patient behaviours and support the development of more robust self-care systems supported by stakeholders and policy at all levels, to work towards an improvement in both oral and general health across the life course.

## Materials and methods

The Dental Policy Lab series began in 2017, bringing together international multi-stakeholder groups of experts to address some of the key issues faced in the battle against dental caries. The first Alliance for a Cavity-Free Future (ACFF)/King's College London Dental Policy Lab (DPL1) addressed the question: 'How do we accelerate a policy shift towards increased resource allocation for caries prevention and control?'^4^ with the second (DPL2) asking: 'How can we create and implement acceptable prevention-based dental payment systems to achieve and maintain health outcomes?'^5^ The Dental Policy Labs set an ambitious agenda of generating system level change through strategic partnerships to create the opportunity for change at several levels.

One of the key outcomes of DPL1 was the recognition of the need to shift public and industry behaviours in order to see improvements towards a cavity-free future. The third Dental Policy Lab (DPL3) took place in 2019 and continued this theme with a 24-hour event focused on the role that dental and oral health industries, governments and dental public health stakeholders have in changing the behaviour of patients and the public. Experts were brought together from across public health, dentistry, governments, global multi-nationals and dental industry start-ups, to address the question: 'How can the oral health and dental industries benefit from enabling positive behaviour in caries prevention and control among patients and the public?' It explored how industry stakeholders could benefit from promoting these changes, with attendees hearing from leading figures in dental psychology, behaviour change and policy to push for the formulation of new ideas through collaborative discussion and innovation.

### Trends in oral health

In understanding how the dental and oral health industries can contribute to achieving a cavity-free future, it is critical to understand the powerful external trends that are likely to shape the next ten years

#### Demographics

Globally, the proportion of populations over the age of 75 is increasing. In addition to an increase in comorbidities and other health conditions (both mental and physical), the shift in demographic is showing generational differences in expectations of oral health, with variations in desire for autonomy and types of people from whom advice is sought.

#### Environment and sustainability

Growing interest in environmental issues and sustainability means both personal and corporate social responsibility (CSR) are likely to play a larger part in decision-making. Awareness of the use of potentially harmful materials and approval for the use of renewable materials also influences healthcare provision.

#### Technology

Increased use of digital technology and the generated data will become a dominant force. Apps, new diagnostic methods and therapeutics will change how diagnosis, monitoring, treatment and even insurance provision will happen. Increased connectivity will influence people's behaviour away from professionals.

#### Workforce

The workforce is shifting, with more dentists, longer careers, shifting gender balance, changing models of employment and changing practice configurations leading to a change in the operational capacity and need of the dental workforce. In the wealthiest markets, corporate dentistry is one of the fastest growing areas, and these shifts are largely driven by consumer demand.^6^ This development in care structure is likely to have a long-term impact on who delivers services, with other healthcare professionals in many areas taking a larger role in detection and prevention services, with dentists shifting to more advanced care.

#### Economics and markets

Currently, most dental remuneration systems are linked to the number of interventions performed, rather than rewarding practitioners for prevention. Increasing allocations for prevention and minimally invasive treatments will be crucial to keeping care affordable. Innovations and technologies that improve efficiency will also gain popularity. To see progress in this area, radically different payment models, incentives and revenue streams must be considered,^5^ such as those currently in trial in France.

#### A changing understanding

**Fig 1:**
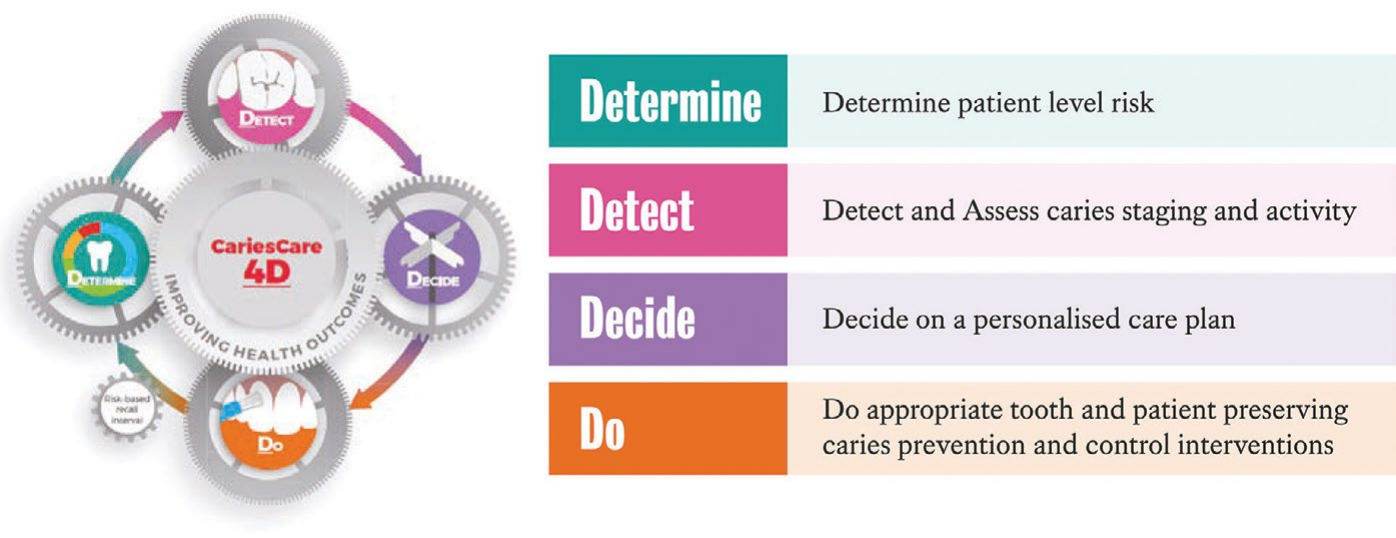
4D model, reproduced with permission from King's College London^11^

A broader understanding among the dental, public health and policy communities of the benefits of a shift towards preventive oral care, both to individuals and to communities/societies, has been developing for a number of years. This was highlighted when in September 2019, the FDI World Dental Federation voted to move towards preventative dental medicine for caries control and to recommend adoption of the concepts of the International Caries Classification and Management System (ICCMS).^7^^,^^8^ The ICCMS has developed over recent years into 'CariesCare 4D'^9^(Fig. 1), a structured and evidence-based approach to caries management for dental practitioners. The process of integrating this system globally is ongoing, with international agreement on implementation process achieved and current roll-out underway.^9^^,^^10^

#### Differing industry structures

The oral health industry is dominated by large, competitive multinationals, often aligned to prevention and able to influence the market. The dental industry is fragmented between companies of differing sizes and relatively few start-ups. The clinical market served by both industries is quite 'conservative' as buyers and is heavily influenced by current payment systems. This can leave companies with their hands tied when it comes to innovation, making introducing new products difficult, and often leaving companies to rely on the push from opinion leaders, universities and policymakers to overcome the barriers preventing change.

## Results

After the third Dental Policy Lab (DPL3) was held, the discussions and takeaway points were summarised in a multi-stakeholder-oriented report, published in 2020.^11^

The ideals and proposals stemming from the discussion can be grouped under four key headings:

- Driving behaviour change
- Operationalising and paying for the ICCMS 4D system
- Influencing regulation and public policy
- Getting oral health onto CSR agendas.

### Driving behaviour change

The DPL3 participants identified four main ways that dental and oral health industries can contribute towards positive behaviour change.

#### Helping understand what is needed for people to change behaviour

The Lab members highlighted the COM-B psychological model of health-related behaviour change. The three COM-B components are:

- Capability - the person must have the physical and psychological skills needed to perform the new behaviour
- Opportunity - the physical availability (for example, products being affordable and accessible) and the social environment (for example, exposure to the new ideas) such that an individual can undertake the new behaviour
- Motivation - the person's conscious (for example, planning and decision-making) and automatic (for example, innate drives, emotional reactions and habits) processes underline the behaviour.

This model demonstrates the complexity of designing interventions for behaviour change. Interventions aimed at transforming capability, motivation and opportunity may operate at the level of public policy, the community or the individual. People may choose not to engage with formal dental care systems because of cost, socioeconomic or other life factors, such as peer group pressure or other social and generational norms or may be blocked from access due to the lack of availability of such systems in certain countries or areas. Achieving behaviour change across a whole population will require all stakeholders to develop approaches aimed at different segments and priorities of the population.

#### Consistent messaging

Inconsistent messages undermine behaviour change. The oral and dental health industries need to be part of a collective effort to give consistent messaging on a range of subjects.

- Understanding the impact of food - restricting sugar intake is key, with particular need during the first two years of life,^12^ to improve early childhood caries, cardiovascular health, obesity and diabetes risks. All messaging should emphasise the behavioural links affecting other aspects of oral health and the common NCD risk factors
- Encouraging good oral hygiene - focus should be on regular brushing twice a day for two minutes with toothpaste containing an appropriate amount of fluoride (or proven alternative). To develop this idea, attempts could be made to normalise brushing outside of the home and to promote the idea that not brushing is not socially acceptable
- Sources of care and support - support from other healthcare professionals could also be advised. People must be alerted to misleading and non-evidence-based advice available from non-traditional sources. Families and carers of older people should receive clear messaging on the need for regular, tailored care for those in their charge, and advice on how to achieve it
- A shift from treatment to prevention - the benefits of shifting to prevention should be emphasised to and by all stakeholders. The individual, societal (cost-saving) and global (environmental) benefits of preventive dentistry should be promoted at all opportunities.

#### Encouraging behaviour change

Widespread and sustainable change requires a shift in social perception away from 'it's just caries'. Messaging needs to be complemented by supportive public policies and laws.

Three ways were identified for dental and oral health industries to push to support this change:

- Campaigns to grab the attention of the public - raising awareness for caries should be part of broader messaging about the importance of oral health, blending positive and negative messaging. Links to other behaviour changes, such as the promotion of breastfeeding or reducing smoking/alcohol consumption, should be taken advantage of
- Public-private partnerships to put in place practical support - public-private partnerships can play a powerful role in bringing about positive social impacts by designing and operating the resources with and within the communities they help. They can drive innovation, leverage resources, bring efficiencies, and introduce technology and best practices. They create an emotional connection through authentic, community-driven messaging. They can bring in other stakeholders and public as champions, influencers and donors, and offer incentives
- Product and/or theme placement to maintain awareness and reinforce key messages - encouraging and supporting the use of product and/or theme placement in movies and other media could help reinforce key messaging. Industries could invest more into online communication through connecting with influencers, key brands and media channels.

#### Engaging patients with technology

In a world with ever-developing technology, there are multiple opportunities for increased engagement through technology platforms. Industries must improve and develop their online communication, utilising current trends to reach wider audiences. One example of this potential opportunity would be for the creation of an app to provide individualised advice to patients and the public, utilising individualised risk profiling and 'predictive preventing plans', offering direction and advice to sites and resources, with the potential to link to dental practices for monitoring.

### Operationalising and paying practitioners for using the ICCMS/CariesCare International 4D system

Support from all stakeholders is crucial in facilitating the continued world-wide roll-out of the free-to-use ICCMS/CariesCare International 4D model. The promise of reimbursement for dental practitioners for the application of preventive treatment plans and remuneration of each of the 4Ds in practice is critical to getting preventively oriented approaches fully adopted into practices.

To achieve this, ideally, a dental practice would have:

- New types of providers and a mix of other practitioners as well as dentists
- Patients organised by risk
- Remuneration divided across the patient base with incentives promoting evidence-based protocols for individual patients and achieving health outcomes.

**Fig 2:**
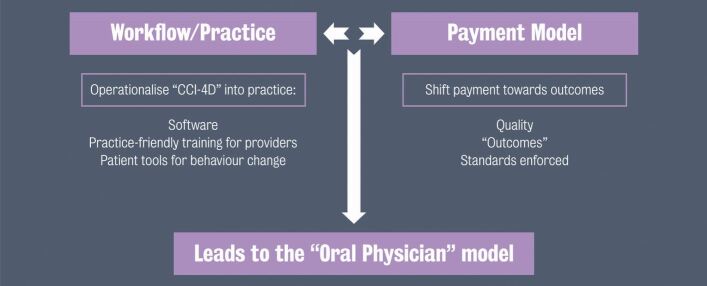
Workflow practice model, reproduced with permission from King's College London^11^

The development of the current practice model in this way would lead to a more holistic 'oral physician' model of dentistry (Fig. 2).

If actioned correctly, this approach would offer further benefits to practitioners, including more time to expand their patient base and for 'added value' work such as aesthetics, the diversification of dental care across practitioner specialties into wider oral health and towards more general health hubs, and potentially the avoidance of litigation through accurate recording of audit trails of advice and care to support prevention.

There is opportunity for industry to provide the inputs to support this new model, such as:

- The technology and software for comprehensive diagnostics, risk assessment and decision support
- Training, education and support to introduce the solution across the profession
- Sales, financing, project management and implementation skills to help individual practices acquire the skills
- The tools that inform and engage patients in their own behaviours.

### Influencing regulation and public policy

Many factors in the dental and oral health markets are outside of industry control. Systems are not currently built to support innovation or action around preventive care. Despite this, increasing opportunity to influence the policymakers and regulatory groups concerned is being afforded to industry stakeholders. To see development in the policy landscape to allow for a preventive shift in dental care, steps must be taken by all stakeholders to push towards the following:

- Integrating and aligning upstream, midstream and downstream - there needs to be a link between 'upstream' public health efforts around caries prevention with the 'midstream' clinical practices and the 'downstream' patient behaviours
- Making preventative care accessible and affordable - the participants proposed that in the UK, VAT on toothpaste and other necessary oral hygiene products should be removed as part of an effort to make oral health affordable to all
- Supportive regulation for innovation - industry needs help from public policy to create a harmonised regulatory environment around prevention-based approaches, supported by insurers and payers and endorsed by health departments and Chief Dental Officers (CDOs). To achieve this would give companies a level playing field to innovate on and encourage further development.

### Getting oral health onto CSR agendas

The opportunity exists to influence companies to strive to improve oral health through their CSR work - to create a global push, spearheaded by CDOs, to work alongside industry and to gently influence the direction of CSR programmes by providing aligned messaging, which can be locally adapted to encourage the adoption of appropriate programmes. This process may be best facilitated by international organisations, utilising networks of CDOs, collaborators and charity structures to aid in the development of these programmes most effectively.

The benefits of achieving this are wide; for industry, the creation of a targeted CSR agenda which could help connect and scale up pilot oral health programmes, link initiatives into wider healthy life programmes and tie oral health to sustainability; for governments, the benefit would come through the promotion of key public health goals; for the public, there would be a common agenda working against a disease that affects nearly 100% of the adult population.

Following on from discussions at DPL3, the 'making cavities history' initiative, developing global policies for integrated caries prevention, was initiated.^13^

## Conclusions

The Policy Lab methodology is a highly effective method to guide and influence high-level discussions concerning policy developments. Following DPL3, a Lab report was published, targeted towards informing industry and policy stakeholders of the outcomes of the meeting. As previously, the development of an infographic outlining the key elements of the report has proved a hugely useful tool for dissemination and communication (Appendix 1). The discussions included in this Lab have been taken forward by stakeholders in multiple countries, and the work of DPL3 will continue to feed into the direction of the newly formed 'Dental Policy Lab Network', overseen by the ACFF and aiming to build further upon the identified steps for development drawn from across the three Dental Policy Labs.

**Figure:**
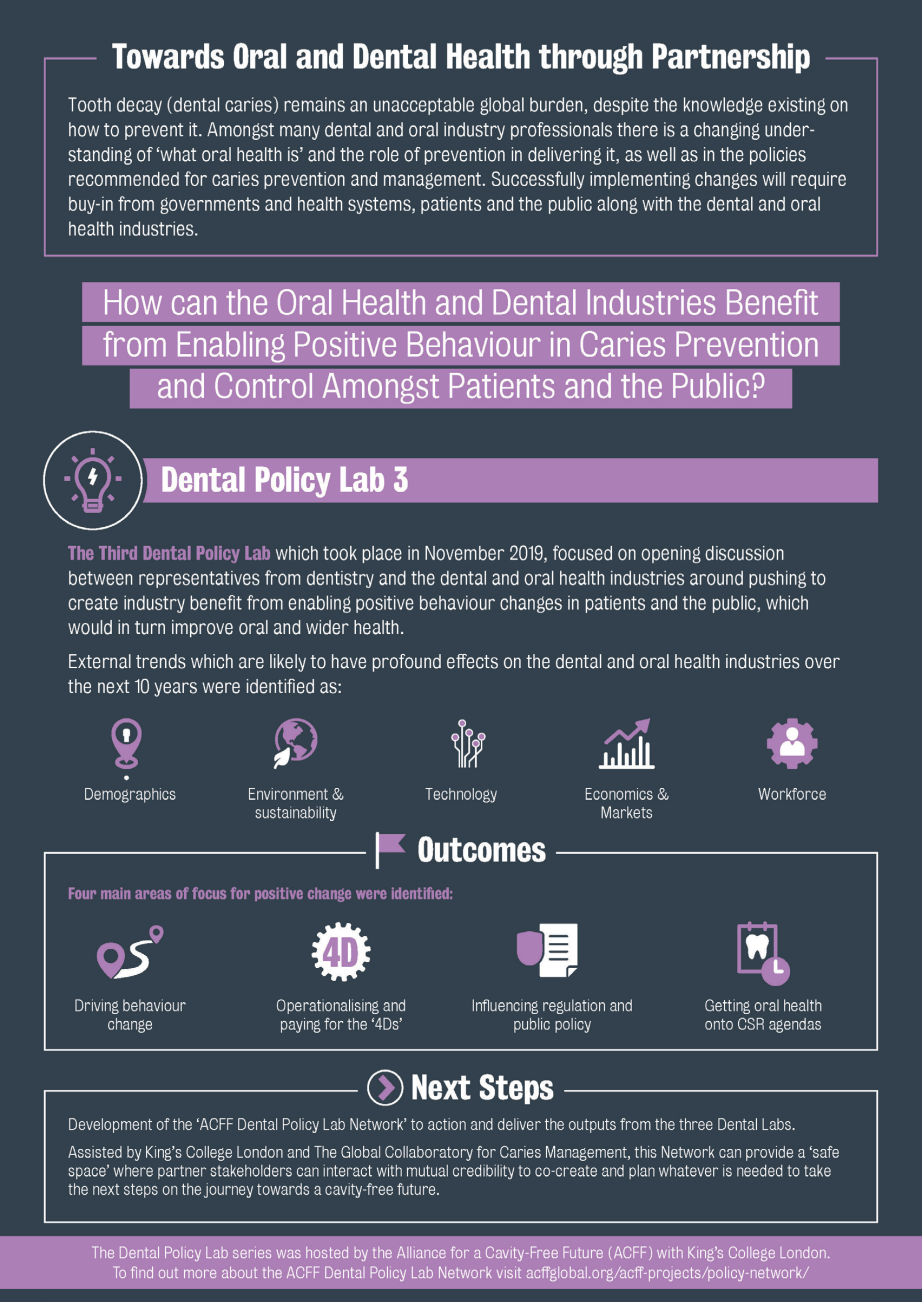
***Appendix 1*** Dental Policy Lab 3 overview infographic^11^
